# Implications for Cardiac Function Following Rescue of the Dystrophic Diaphragm in a Mouse Model of Duchenne Muscular Dystrophy

**DOI:** 10.1038/srep11632

**Published:** 2015-06-26

**Authors:** Corinne A. Betts, Amer F. Saleh, Carolyn A. Carr, Sofia Muses, Kim E. Wells, Suzan M. Hammond, Caroline Godfrey, Graham McClorey, Caroline Woffindale, Kieran Clarke, Dominic J. Wells, Michael J. Gait, Matthew J. A. Wood

**Affiliations:** 1 Department of Physiology, Anatomy and Genetics, University of Oxford, South Parks Road, Oxford, UK, OX1 3QX; 2 Medical Research Council, Laboratory of Molecular Biology, Francis Crick Avenue, Cambridge, CB2 0QH, UK; 3 AstraZeneca R&D, Discovery Safety, Drug safety and Metabolism, Alderley Park, Macclesfield, SK10 4TG, UK; 4 Department of Comparative Biomedical Sciences, Royal Veterinary College, Royal College Street, London, NW1 0TU, UK

## Abstract

Duchenne muscular dystrophy (DMD) is caused by absence of the integral structural protein, dystrophin, which renders muscle fibres susceptible to injury and degeneration. This ultimately results in cardiorespiratory dysfunction, which is the predominant cause of death in DMD patients, and highlights the importance of therapeutic targeting of the cardiorespiratory system. While there is some evidence to suggest that restoring dystrophin in the diaphragm improves both respiratory and cardiac function, the role of the diaphragm is not well understood. Here using exon skipping oligonucleotides we predominantly restored dystrophin in the diaphragm and assessed cardiac function by MRI. This approach reduced diaphragmatic pathophysiology and markedly improved diaphragm function but did not improve cardiac function or pathophysiology, with or without exercise. Interestingly, exercise resulted in a reduction of dystrophin protein and exon skipping in the diaphragm. This suggests that treatment regimens may require modification in more active patients. In conclusion, whilst the diaphragm is an important respiratory muscle, it is likely that dystrophin needs to be restored in other tissues, including multiple accessory respiratory muscles, and of course the heart itself for appropriate therapeutic outcomes. This supports the requirement of a body-wide therapy to treat DMD.

Cardiac and respiratory complications are the leading cause of death amongst Duchenne muscular dystrophy (DMD) patients^1^,^2^. DMD is a debilitating muscle wasting disorder, caused by mutations/deletions in the *Dmd* gene which disrupts the reading frame and prevents the translation of dystrophin protein^3^,^4^,^5^. Dystrophin is an important sarcolemmal protein which stabilises the sarcolemma^6^, and thus dystrophin-deficient muscles are particularly susceptible to contraction-induced damage and muscle fibres are replaced with fibrous connective tissue and fat rendering the muscle ineffective^7^,^8^. Indeed it is the deposition of fibrous tissue and further deterioration of the diaphragm which contributes significantly to respiratory insufficiency^9^. In addition, impaired respiration compromises cardiac function due to increased pulmonary hypertension which eventually leads to dilated cardiomyopathy^10^. Other symptoms of cardiomyopathy in DMD patients include tachycardia, electrocardiogram (ECG) abnormalities, left ventricle thickening (hypertrophy) and a decrease in fractional shortening^11^,^12^. It should be noted that splice-switching oligonucleotide (SSO) therapies currently undergoing clinical trial, namely phosphorodiamidate morpholino oligomer and 2’O methyl phosphorothioate oligonucleotides, are not capable of restoring dystrophin in the heart^13^,^14^,^15^,^16^. Therefore it is essential to address the contribution of other important muscles, such as the diaphragm to cardiorespiratory function.

Improvement in cardio-respiratory function in DMD patients would enhance quality of life and prevent fatal complications. Two independent studies have proposed that the restoration of dystrophin in the diaphragm of DMD mouse models has a profound beneficial effect on respiratory^17^ and cardiac function^18^. If this were indeed the case, it would lessen the requirement for treating the heart, which is an especially difficult tissue to target. However, neither study completely answers the question as to what extent restoring dystrophin to the dystrophic diaphragm is able to improve cardiac function. The study which assessed respiratory function showed that specific restoration of dystrophin in the diaphragm using a helper-dependent adenovirus vector, increased tidal volume and improved compensatory hyperpnea^17^. However, neither cardiac function nor cardiac histopathology were assessed in this study. The second study did assess cardiac function, but dystrophin was restored in multiple muscles including skeletal muscles and accessory respiratory muscles and therefore the contribution of a rescued diaphragm exhibiting physiological improvement could not be assessed^18^.

To address the role of the diaphragm, we predominantly restored dystrophin in the diaphragm of *mdx* mice using an exon skipping approach *via* use of SSO-peptide conjugates, and measured the effect of the rescued diaphragmatic dystrophin expression and function on cardiac function, using MRI. Increased activity has also been shown to contribute to a progressed cardiac phenotype^19^, and therefore we also assessed whether restoration of dystrophin in the diaphragm is capable of rescuing cardiac function in the presence of forced exercise. In short, the intraperitoneal route of administration resulted in substantial dystrophin restoration to the diaphragm and abdominal wall muscles (due to proximity of administration site), and low to negligible levels of dystrophin protein in sternocleidomastoid and intercostal muscles. The restoration of dystrophin in the diaphragm markedly improved diaphragmatic physiology, notably specific force and resistance to eccentric exercise induced force drop, and reduced pathophysiology of this muscle, but did not have a beneficial effect on cardiac function or cardiac pathophysiology, with or without exercise. In addition, there was significantly less dystrophin restored to the diaphragm of treated, exercised *mdx* mice.

## Results

### Intravenous Peptide-PMO administration Restores Dystrophin in Additional Respiratory Muscles

**Figure 1:**
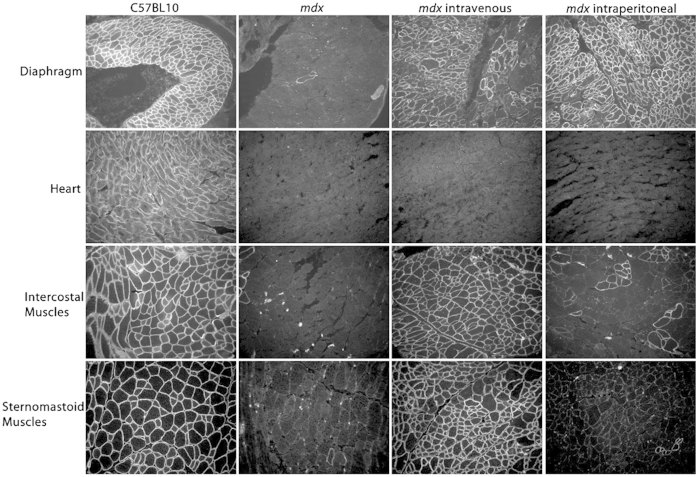
Immunohistochemical staining of dystrophin comparing B-PMO treatment following intravenous and intraperitoneal administration. Representative images of dystrophin staining for diaphragm, heart, intercostal muscles and sternomastoid muscles are shown. C57BL/10 and untreated *mdx* muscles were compared to *mdx* mice treated by intravenous and intraperitoneal routes of administration. For treated groups, mice received a single 19 mg/kg injection. Figure illustrates restoration of dystrophin in diaphragm and respiratory muscles, the intercostal and sternomastoid muscles, when B-PMO was administered intravenously. However dystrophin was only prevalent in diaphragm following intraperitoneal administration.

It has previously been demonstrated that a single intravenous administration of phosphorodiamidate morpholino oligonucleotides (PMO), conjugated to an arginine-rich (RXRRBR)_2_ peptide (B-peptide), improves cardiac function in *mdx* mice^18^,^20^. The authors attributed this to the restoration of dystrophin to the diaphragm^18^. However, it is known that intravenous administration of peptide-PMO conjugates results in highly efficient body-wide skeletal muscle delivery and dystrophin restoration^21^, whereas intraperitoneal administration results predominantly in diaphragmatic dystrophin restoration^22^. It is therefore likely that the intravenous route of administration restored dystrophin in other muscles such as respiratory muscles. To determine any differences in dystrophin restoration between the two administration routes, a single 19 mg/kg dose (as used in Crisp *et al.*^18^) was delivered either intravenously *via* the tail vein or *via* the intraperitoneal route, and tissues were harvested 3 weeks later (Fig. 1). Intravenous administration of B-PMO resulted in widespread and high levels of dystrophin restoration in major respiratory muscles including the diaphragm, intercostal and sternomastoid muscles. As expected, the intraperitoneal administration resulted in marked dystrophin restoration in the diaphragm, but with very low levels of dystrophin in the intercostal and sternomastoid muscles, thus demonstrating the marked disparity between the two delivery routes. Neither administration route resulted in dystrophin restoration in heart at the dose used.

### Substantial Dystrophin Restoration in Diaphragm Following Intraperitoneal Administration

**Figure 2:**
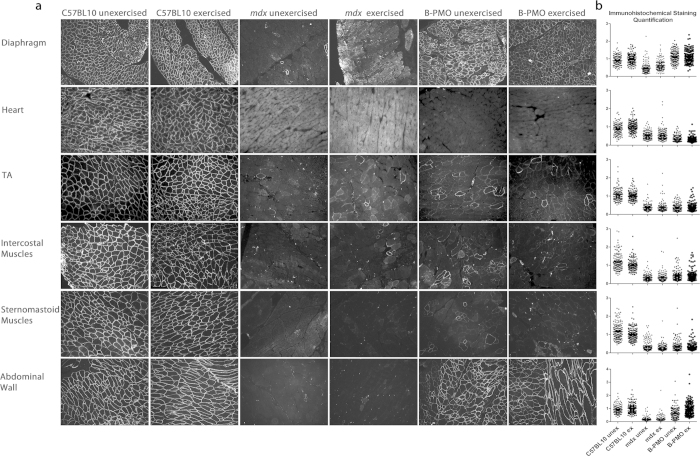
Immunohistochemical staining and quantification of dystrophin following B-PMO treatment delivered via intraperitoneal route of administration. (**a**) Representative images of immunohistochemical staining of dystrophin protein in exercised and unexercised C57BL/10, *mdx* and B-PMO treated *mdx* mice showing the diaphragm, heart, *tibialis anterior*, intercostal muscles, sternomastoid muscles and abdominal wall. Tissues were harvested 3 weeks after B-PMO administration (19 mg/kg administration). (**b**) Dystrophin immunohistochemical staining quantification following B-PMO treatment in *mdx* mice. Dystrophin expression was calculated relative to laminin co-stain (120 regions of interest per slide), and normalised relative to the C57BL/10 unexercised group. The box plots show the normalised relative intensity values for each region of interest, and the distribution pattern thereof. N = 4 for each cohort.

**Figure 3:**
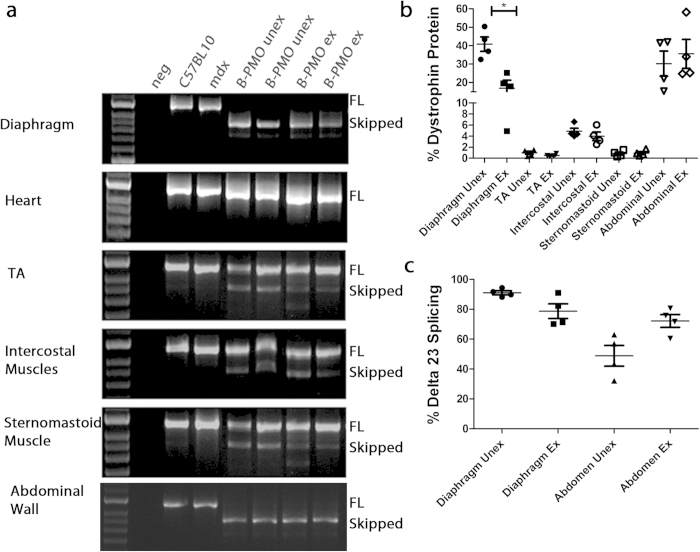
Dystrophin splicing and protein restoration in B-PMO treated mice compared to *mdx* untreated and C57BL/10 control groups. (**a**) Representative images of reverse-transcriptase (RT) PCR illustrating exon 23 exclusion in diaphragm, heart, *tibialis anterior* (TA), intercostal muscles, sternomastoid muscles and abdominal wall of B-PMO treated cohort. The top band represents full length dystrophin (FL) and the lower band represents the skipped transcript (Skipped). (**b**) Protein quantification of western blots in diaphragm, TA, intercostal and sternomastoid muscles of B-PMO treated mice. 10–15 μg of protein was loaded and quantified relative to vinculin loading control. Average values and SEM are as follows: diaphragm unexercised: 41% SEM 3.9, exercised: 17% SEM 4.3; TA unexercised: 1% SEM 0.3, exercised: 0.5% SEM 0.1); intercostal unexercised: 5% SEM 0.6, exercised: 4% SEM 0.8; sternomastoid muscles unexercised: 0.8% SEM 0.3, exercised: 0.9% SEM 0.3; and abdominal wall unexercised: 30% SEM 6.8, exercised: 36% SEM 7.8. Note: no protein was detected in hearts of B-PMO treated mice (See Supplementary Fig. S2 online). (**c**) RT-qPCR was also performed on diaphragm and abdominal wall samples and confirmed exemplary exon 23 exclusion. N = 4 for each cohort. Statistical significance was determined using Students T-test (p < 0.05).

Accessory respiratory muscles may also facilitate respiratory function, and therefore the intravenous administration route does not demonstrate the specific impact of a corrected diaphragm in DMD. Therefore, building on the comparative route of administration experiment (Fig. 1), a detailed study quantifying dystrophin restoration levels and cardiac function following intraperitoneal administration of B-PMO, was initiated (21 week old mice administered dose of 19 mg/kg; modelled closely to Crisp *et al.*^18^). For exercised groups, mice were run the following day, and every second day thereafter for 3 weeks (10 bouts of exercise in total; Supplementary Fig. S1 online). Tissues were harvested at 24 weeks of age. Exon skipping and dystrophin protein restoration in the diaphragms of B-PMO treated *mdx* mice were assessed using routine techniques, namely immunohistochemical staining, RT-PCR, quantitative real time-PCR (RT-qPCR) and western blotting. Additional tissues assessed for dystrophin restoration included the heart, *tibialis anterior*, abdominal wall, intercostal muscles and sternomastoid muscles. Immunohistochemical staining for dystrophin in the B-PMO treated cohorts revealed extensive dystrophin expression in the diaphragm (Fig. 2a), which is illustrated by the box plots showing the normalised relative intensity values for each region of interest. The distribution pattern of the B-PMO treated cohorts correlates with the C57BL/10 cohorts (Fig. 2b). Dystrophin expression was very low in the *tibialis anterior*, intercostal and sternomastoid muscles and completely absent in the heart (Fig. 2; similar distribution patterns to mdx). Intraperitoneal administration also resulted in dystrophin expression in the abdominal wall muscle, which is not surprising given the proximity of the administration site. The RT-PCR and western blots revealed complete exon skipping of dystrophin (Fig. 3a) and substantial dystrophin protein restoration in the diaphragms of B-PMO treated mice (unexercised 41%, exercised 16%; Fig. 3b and Supplementary Fig. S2 online). Splicing efficiency in the diaphragms of B-PMO treated mice was confirmed by RT-qPCR which resulted in 91% and 79% exon 23 exclusion in unexercised and exercised mice, respectively (Fig. 3c). Dystrophin splicing levels in the *tibialis anterior*, intercostal and sternomastoid muscles were low, with approximately 1% dystrophin protein restoration in the *tibialis anterior* and sternomastoid muscles, and 4% restoration in intercostal muscles. Dystrophin splicing and protein restoration was absent in the hearts of B-PMO treated mice. High dystrophin splicing levels (unexercised 49%, exercised 72%; Fig. 3c) and marked protein restoration (unexercised 30%, exercised 36%; Fig. 3b) were also observed in the abdominal wall muscles of B-PMO treated mice.

Interestingly there was a significant disparity in dystrophin protein restoration (measured by western blot; Fig. 3b) in the diaphragms of unexercised and exercised B-PMO treated *mdx* mice (p < 0.05). A reduction in dystrophin splicing was also observed in exercised treated mice when considering RT-qPCR data (i.e. 91% versus 79% exon 23 exclusion).

### Rescue of Diaphragmatic Dystrophin Does Not Improve Cardiac Function

**Figure 4:**
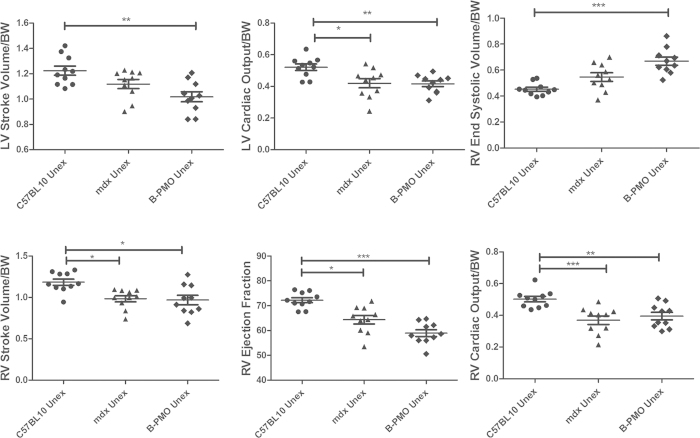
Cardiac function parameters measured by cine-MRI in unexercised mouse cohorts. Scatter plot graphs illustrating individual mouse values, mean and standard error of the mean (SEM) for left ventricular (LV) and right ventricular (RV) functional parameters relative to body weight. Note: The exception to this was the ejection fraction which was represented as a percentage (it is a volumetric fraction). B-PMO and *mdx* cohorts exhibited worse cardiac function than C57BL/10 mice. Statistical significance was determined using ANOVA followed by Tukey post-hoc test or Games-Howell post-hoc test to correct for variance heterogeneity. (***p < 0.001, **p < 0.01, *p < 0.05). N = 10 for each cohort.

Mice underwent MRI at 24 weeks of age. All cardiac parameters were normalised to weight (with the exception of the ejection fraction (EF) which is the volumetric fraction of blood pumped from each ventricle after each heartbeat and is independent of weight). Cardiac function of the unexercised *mdx* and unexercised B-PMO treated cohorts was compared with that of their C57BL/10 counterpart (n = 10 for each cohort). The unexercised *mdx* cohort revealed lower left ventricular cardiac output (LV CO) and changes in a number of right ventricle (RV) measurements, relative to unexercised C57BL/10 mice (Fig. 4). RV stroke volume (SV) was lowered, with subsequent reduction in EF and CO suggesting impaired contractility. Similarly, the unexercised B-PMO treated mice also exhibited low LV SV and CO, and changes in multiple RV parameters relative to unexercised C57BL/10 (Fig. 4 and Table 1). This indicates that significant restoration of dystrophin protein in the diaphragm and abdominal wall muscles (B-PMO unexercised mice) did not improve cardiac function.

Exercised cohorts were also included in the study to determine whether dystrophin in the diaphragm can prevent deterioration of the cardiac phenotype in *mdx* mice, in the event of exercise. These mice underwent 10 bouts of forced exercise over the course of 3 weeks. The exercise had a significant effect on the untreated *mdx* mouse hearts for a number of cardiac measurements, as determined by Two-way ANOVA (see Table 1 for interaction effects). The *mdx* exercised cohort revealed lowered LV SV and increased RV end systolic volume with consequential decrease in RV EF (see Table 1 for significance between unexercised and exercised *mdx* cohorts). This suggests that exercise resulted in further deterioration of RV contractility in *mdx* mice. In contrast, the C57BL/10 and B-PMO treated cohorts were largely unchanged by exercise (see Table 1 for significance relative to exercised counterpart).

**Figure 5:**
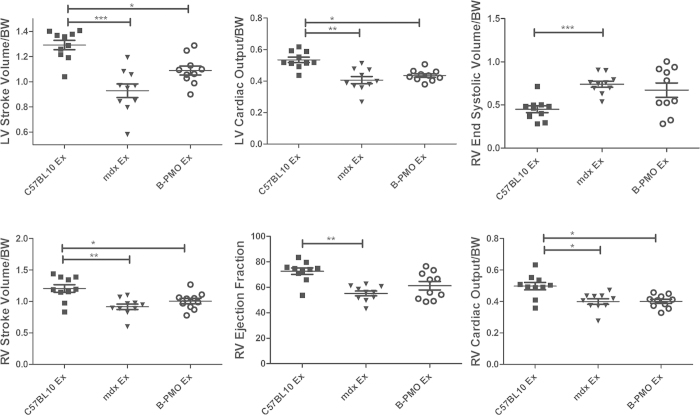
Cardiac function parameters measured by cine-MRI in exercised mouse cohorts. Scatter plot graphs illustrating individual mouse values, mean and standard error of the mean (SEM) for left ventricular (LV) and right ventricular (RV) parameters relative to body weight. Note: The exception to this is the ejection fraction which is represented as a percentage (it is a volumetric fraction). Cardiac function in B-PMO and *mdx* was worse than C57BL/10 mice. Statistical significance was determined using ANOVA followed by Tukey post-hoc test or Games-Howell post-hoc test to correct for variance heterogeneity. (***p < 0.001, **p < 0.01, *p < 0.05). N = 10 for each cohort.

Direct comparison of the *mdx* exercised cohort with its C57BL/10 counterpart revealed significant changes in LV SV and LV CO (Fig. 5 and Table 1). The *mdx* group also revealed pronounced changes in a number of RV parameters relative to the C57BL/10 counterpart. Although the B-PMO treated cohort did not deteriorate with exercise, this group revealed similar cardiac function parameter values to the *mdx* exercised cohort (LV SV, LV CO lower, RV SV and RV CO lower than the C57BL/10). Therefore the B-PMO treatment and rescue of dystrophin expression in the diaphragm and abdominal wall muscles did not protect heart function in the event of exercise.

### Restoration of Diaphragmatic Dystrophin Markedly Improves Muscle Function

**Figure 6:**
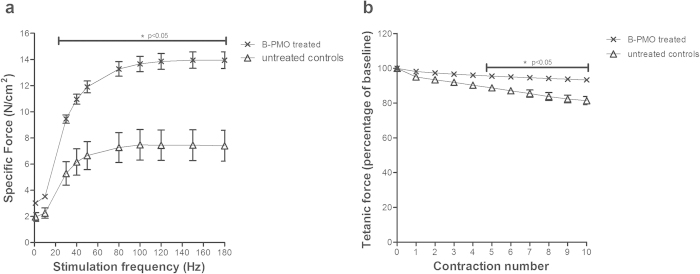
*In vitro* assessment of muscle function in B-PMO treated diaphragms. *Mdx* mice received a single intraperitoneal injection of B-PMO and age, sex and litter-mate matched *mdx* mice were used as untreated controls. Three weeks post-treatment diaphragms were removed and muscle function was assessed *in vitro*. (**a**) Force-frequency curves showing specific isometric force production in both groups (N/cm^2^). A significant improvement in tetanic force was observed in diaphragm strips of B-PMO treated mice compared to untreated controls (stimulation frequencies 30-180Hz). In addition, an 88% improvement in maximal specific isometric force production was noted in B-PMO treated diaphragms (13.94 ± 0.69 N/cm^2^) compared to the control group (7.4 ± 1.26 N/cm^2^). (**b**) Using an eccentric contraction protocol, B-PMO treated diaphragms had significant protection against eccentric contraction-induced muscle damage from eccentric contraction number 4 through to 10. A final tetanic force drop of 6.7 ± 0.87% compared to baseline was noted in B-PMO treated diaphragms, whilst control diaphragms exhibited a greater force loss of 18.6 ± 2.55%. Statistical significance was determined using Two-way repeated-measure ANOVA with Bonferroni’s post-hoc test, (*p = <0.05), N = 7/8.

To assess the functional effects of B-PMO administration on the diaphragm, we treated 21-week old male *mdx* (n = 7) with a single intraperitoneal injection of B-PMO (19 mg/kg). Age and litter matched male *mdx* mice were used as controls (n = 8). Three weeks post-treatment we evaluated the force-frequency relationship between the two groups. A significant improvement in specific force (N/cm^2^) was noted over a range of stimulation frequencies (30 Hz-180 Hz) in the B-PMO treated mice (Fig. 6a). In addition, a single dose of B-PMO improved maximal specific isometric force by 88% (13.94 ± 0.69 N/cm^2^) compared to non-treated controls (7.4 ± 1.26 N/cm^2^). Using a muscle eccentric contraction protocol with 10% stretch, we measured resistance to eccentric contraction-induced muscle damage. Diaphragms from B-PMO treated mice exhibited significant protection against eccentric contraction-induced muscle damage, from contraction number four to ten, in contrast to untreated controls (Fig. 6b). A final tetanic force loss of 6.7 ± 0.87% compared to baseline was observed in the B-PMO treated groups, unlike the control mice which exhibited an 18.6 ± 2.55% force drop from baseline. Thus a single intraperitoneal administration was sufficient to markedly improve diaphragm function.

### Pathophysiological Improvement in Dystrophin-Restored Diaphragm Does Not Ameliorate the Cardiac Phenotype

**Figure 7:**
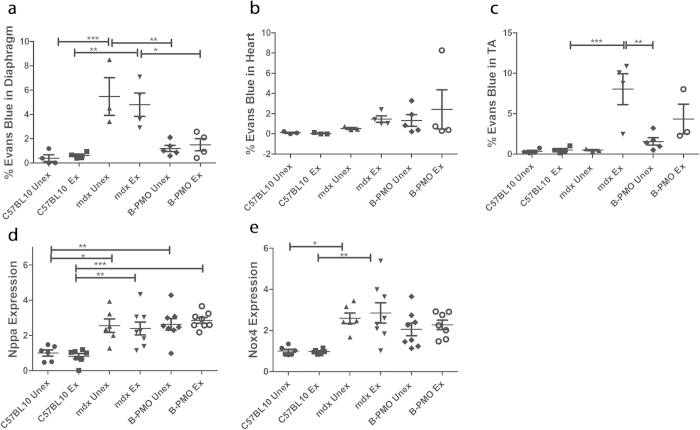
Quantification of Evan’s blue dye leakage in diaphragm, heart and *tibialis anterior* muscles and gene expression of markers for injury in the hearts of exercised and unexercised C57BL/10, *mdx* and B-PMO treated mice. (**a-c)** Evans blue leakage was quantified by calculating the surface area of stained cells relative to total surface area of section. Scatter plot graphs of Evans blue staining illustrating individual mouse values, mean and standard error of the mean (SEM). Evans blue leakage was pronounced in diaphragm of *mdx* mice, and *tibialis anterior* of exercised *mdx* and B-PMO mice. No statistical significance between cohorts for heart. N = 3–4 for each cohort. Quantitative real time (RT-q)PCR for the expression of *Nppa* (**d**) and *Nox4* (**e**) relative to *Ywhaz* in heart, normalised to C57BL/10 unexercised cohort. Expression of *Nppa* was elevated in B-PMO and *mdx* cohorts, and *Nox4* elevated in *mdx* unexercised cohort. N = 8 for each cohort. Statistical significance was determined using one-way ANOVA, Tukey post-hoc test (***p < 0.001, **p < 0.01, *p < 0.05).

Substantial restoration of dystrophin in the diaphragm also reduced diaphragmatic pathophysiology, as shown by reduced Evans blue dye (EBD) leakage into the muscle. EBD is a tracer used to determine the extent of sarcolemmal damage as a result of the lack of dystrophin in muscle^23^,^24^. EBD leakage is markedly elevated in *mdx* mice, particularly if exercised. The surface area of EBD infiltration into the diaphragms of the *mdx* unexercised and exercised cohorts were both markedly greater than the unexercised and exercised C57BL/10 and B-PMO cohorts (Fig. 7a). The B-PMO treated mice exhibited a near normalised EBD profile, which was anticipated given the substantial amount of dystrophin in the diaphragm, thus improving membrane stability and preventing the infiltration of EBD into tissue.

EBD leakage into the *tibialis anterior* of the *mdx* exercised mice was significantly higher than all other cohorts with the exception of the B-PMO exercised counterpart (*mdx* exercised cohort: average of 8% EBD infiltration; Fig. 7c). The B-PMO exercised cohort also exhibited marked elevation in EBD infiltration however to a lesser extent then the *mdx* exercised cohort. This confirms that the intraperitoneal administration did not successfully restore dystrophin in peripheral muscles such as the *tibialis anterior*, to substantially reduce pathophysiology.

Whilst there was no significant EBD leakage into the hearts of any cohort, it should be noted that the *mdx* exercised, B-PMO unexercised and B-PMO exercised cohorts all exhibited slightly elevated levels of infiltration. Indeed one of the B-PMO exercised mice displayed marked EBD leakage (see Fig. 7b and Supplementary Fig. S3 online). In addition, there was no reduction in the expression of genes indicative of cardiac damage. RT-qPCR was carried out on heart tissue to assess the expression of markers for haemodynamic overload and oxidative stress^25^,^26^,^27^, *Nppa* and *Nox4* respectively relative to *Ywhaz* (validated cardiac housekeeping gene). *Nppa* expression in *mdx* and B-PMO treated hearts was higher than their C57BL/10 counterparts (in unexercised and exercised mice; Fig. 7d). *Nox4* expression was significantly raised in the *mdx* unexercised cohort. The *mdx* exercised and B-PMO cohorts were approximately 2-fold up-regulated (Fig. 7e). The elevated expression levels of these genes show that restoration of dystrophin in the diaphragm has not improved the cardiac phenotype of mice with dystrophic hearts.

## Discussion

Our aim was to discern the effect of dystrophin restoration in the diaphragm on heart function in the *mdx* mouse model of DMD. A single intraperitoneal injection resulted in significant and widespread dystrophin in the diaphragm, with insignificant expression in other skeletal muscles, with the exception of the abdominal wall. However, whilst there was some reduction in diaphragmatic pathophysiology (reduced sarcolemmal damage), and marked improvement in diaphragm muscle function, dystrophin restoration in this respiratory muscle did not benefit cardiac function or pathophysiology, with or without exercise. In addition, restoration of dystrophin in the abdominal wall did not assist cardiac function either. This was surprising, as the diaphragm is the major respiratory muscle, and it was anticipated that restoring dystrophin would reduce vascular resistance and pulmonary hypertension^10^,^17^,^28^.

The outcome of this investigation contrasts with that of the study by Crisp *et al.*^18^, which utilised the same peptide-PMO conjugate. The distinct difference between these two studies is the delivery route i.e. intravenous versus intraperitoneal. Intravenous delivery restored high levels of dystrophin in the diaphragm, but also led to widespread expression in multiple other muscles including multiple respiratory muscles, as confirmed in this study (Fig. 1). Perhaps the intercostal and sternocleidomastoid respiratory muscles play a more important role than previously anticipated in overall respiratory function in the *mdx* mouse. Indeed the intercostal muscles are important for adequate inspiratory (external intercostals) and expiratory (internal intercostals) function^29^. Dystrophin is also present in smooth muscle and vascular endothelial cells in the microvasculature, where it regulates eNOS and thus NO handling^28^. However, in *mdx* mice, dystrophin is absent in vascular endothelial cells^30^,^31^,^32^ and the tunica media of blood vessels^33^, which may contribute to impaired NO signalling and therefore vasodilatory response^28^. The finding of dystrophin positive cells in blood vessels of liver sections has been demonstrated following very high and chronic dosing of PMO^34^. Perhaps intravenous administration with peptide-PMO restored dystrophin in the microvasculature, improving vasoregulation and thereby reducing vascular resistance. This theory of poor vasoregulation is supported by emerging evidence that *mdx* mice function under hypoxic conditions^35^,^36^.

Conversely, in the present study when intraperitoneal injection was used, dystrophin may not have been present in most respiratory muscles or the microvasculature. Therefore inspiratory and expiratory function may have been impaired due to the absence of dystrophin in respiratory muscles, particularly the intercostal muscles. Alternatively the absence of dystrophin in the vasculature may have led to poor vasoregulation, thus aggravating vascular resistance.

The increase in workload during exercise interestingly resulted in a reduction of dystrophin protein in the diaphragm. This may be due to several factors such as the inability to fully protect the muscle from increased workload, a potentially less stable transcript, or that the exercise regimen was initiated prior to the administration having a physiological effect. In addition, this emphasises the importance of monitoring active patients who may require more frequent treatment administrations. This is also important given that current oligonucleotide chemistries undergoing clinical trial have demonstrated low systemic delivery and no restoration of dystrophin in cardiac muscle^13^,^14^,^15^,^16^. This highlights concerns pertaining to increased activity in patients.

The data demonstrate that restoration of dystrophin in the diaphragm markedly improved muscle function and reduced pathophysiology, yet did not have a beneficial effect on cardiorespiratory function. The diaphragm is a major respiratory muscle and therefore a major muscle to target. However restoration of dystrophin in this muscle alone is only likely to confer cardiorespiratory benefit when coupled with restoration in respiratory muscles and, of course, in the heart itself. Indeed there are peptide-PMO modalities that are capable of targeting the heart such as Pip-PMOs^37^,^38^. In short, this study shows that targeting a single muscle or organ to treat the systemic disease DMD is insufficient. Instead it emphasises the importance of a body-wide treatment and work to find a therapy and delivery strategy that will restore dystrophin in particularly difficult to target organs.

## Materials and Methods

### Synthesis of peptide-PMO conjugates

The PMO sequence (5’-GGCCAAACCTCGGCTTAC CTGAAAT-3′) was purchased from Gene Tools LLC. B peptide (RXRRBRRXRRBRXB) was conjugated to PMO at the 3′ end *via* an amide linkage by the method previously described^37^. The B-PMO conjugate was purified by HPLC and was analysed by MALDI-TOF mass spectrometry. Peptide-PMO conjugates were dissolved in sterile water and filtered through a 0.22 μm cellulose acetate membrane before use.

### Animals and intraperitoneal injections

All procedures with the exception of diaphragm physiology were authorized and approved by the University of Oxford ethics committee and UK Home Office (project licence 30/2907, protocol 19b2). These procedures were carried out in the Biomedical Sciences Unit, University of Oxford, in accordance with ‘Laboratory Animal Handbooks NO.14; The Design of Animal Experiments (2010)’. Diaphragm physiology was conducted in the animal facility at the Royal Veterinary College (RVC) under Home Office Licence and with the approval of the RVC Ethics committee. Male *mdx* mice (21 weeks of age) received a single intraperitoneal injection of B-PMO (19 mg/kg) prepared in 0.9% saline solution.

### Exercise Regimen

An Exer 3/6 treadmill (Columbus Instruments, USA) was used for the exercised cohorts. The exercise regimen started at 21 weeks of age (1 day after B-PMO administration for treated cohort) and mice were exercised every second day for 3 weeks (10 exercised bouts in total). Mice were allowed 2 minutes for familiarisation. The exercise regimen for the first 2 exercise days was initiated at 5 m/min and gradually increased in 1 m/min increments to 12 m/min, over a 45 minute period. For the following 2 exercise sessions, the speed was kept between 10 and 12 m/min, and for the remaining sessions (6 bouts) a speed of 12 m/min was maintained for the full 45 minute exercise period.

### Cine-MRI

All mice underwent cardiac cine-MRI at 24 weeks of age, as previously described^39^. Mice were anaesthetised using isoflurane and placed in the supine position into a purpose built cradle. ECG electrodes were inserted into the forepaws of the mouse and the respiration loop was taped across the abdomen. Once the mouse was secured, with a stable ECG measurement, the cradle was lowered into a vertical-bore, 11.7T MR system (Magnex Scientific, Oxon, UK) with a 40 mm birdcage coil (Rapid Biomedical, Wurzburg, Germany). Images were acquired using a Bruker console running Paravision 2.1.1 (Bruker Medical, Ettlingen, Germany). The left and right ventricles were imaged by taking a contiguous stack of cine images in 1 mm increments. Images were analysed using ImageJ software (NIH Image, Bethesda, MD). The epicardial and endocardial borders were outlined using the ImageJ free-hand tool at end-diastole and end-systole.

Following MRI, mice and were sacrificed by CO_2_ inhalation, and muscles and other tissues harvested and snap-frozen in cooled isopentane before storage at −80 °C.

### Diaphragm Physiology

For details of diaphragm muscle physiology, see Supplementary Materials online.

### Evans Blue administration

Three to 4 mice from each cohort received a 1% Evans blue dye (10 μl/gram of mouse; Sigma) intraperitoneal injection. Mice were sacrificed 20 hours later and the TA, diaphragm and heart tissues were collected. These tissues were sectioned (8 μm), soaked in acetone for 10 minutes, rinsed with PBS and mounted. Evans blue infiltration was visualised using a Leitz DM RBE fluorescent microscope (Leica). The entire section was imaged using Axiovision Rel 4.7 Software (Zeiss). Images were manually reassembled and the surface area of Evans blue staining was calculated using the threshold function of ImageJ software.

### Immunohistochemistry and quantification

Transverse sections of tissue samples were sectioned (8 μm thick) and co-stained with rabbit-anti-dystrophin (Abcam) and rat anti-laminin (Sigma) and probed using goat-anti-rabbit IgG Alexa 594 and goat-anti-rat IgG 488 secondary antibodies respectively (Invitrogen). Dystrophin restoration was quantified by acquiring 4 representative images of dystrophin staining and the correlating laminin field for each section, using a Leitz DM RBE fluorescent microscope (Leica) and Axiovision Rel 4.7 Software (Zeiss). Using the ImagePro software (MediaCybernetics), 10 regions of interest were randomly allocated on the laminin image which was overlaid on the corresponding dystrophin image to attain the minimum and maximum fluorescence intensity for each treatment. Data were extrapolated and normalised to C57BL/10 as previously described^37^.

### RT-PCR and RT-qPCR of dystrophin in *mdx* mouse tissues

Total RNA was extracted using TRIzol reagent (Invitrogen) as described in the manufacturer’s instructions. For each RT-PCR reaction, 400 ng of RNA template was used in a 50 μl reverse transcription reaction using One Step RT-PCR Kit (QIAGEN) and gene specific primers as previously described^37^. Two microlitres of cDNA was amplified in a 50 μl nested PCR (QIAGEN PCR kit).

For quantitative analysis of exon skipping levels, 1 μg of RNA was reverse transcribed using the High Capacity cDNA RT Kit (Applied Biosystems) according to manufacturer’s instructions. RT-qPCR analysis was carried out using 25 ng cDNA template and amplified with Taqman Gene Expression Master Mix (Applied Biosystems) on a StepOne Plus Thermocycler (Applied Biosystems). Levels of *Dmd* exon 23 skipping were determined by multiplex qPCR of FAM-labelled primers spanning Exon 20–21 (Assay Mm.PT.47.9564450, Integrated DNA Technologies) and HEX-labelled primers spanning Exon 23–24 (Mm.PT.47.7668824, Integrated DNA Technologies). The percentage of *Dmd* transcripts lacking exon 23 was determined by normalising *Dmd* exon 23–24 amplification levels to *Dmd* exon 20–21 levels.

### RT-qPCR of *Nox4* and *Nppa* in *mdx* mouse hearts

For the RT-qPCR of injury markers, 1 μg of RNA was reverse transcribed using a High Capacity cDNA Synthesis kit (Applied Biosystems). The cDNA was diluted and run using gene specific primers sets (*Nppa* Assay Mm.PT.58.8820983, *Nox4* Assay Mm.PT.58.12973594.g, Integrated DNA Technologies) and TaqMan probe set (Applied Biosystems) on the StepOne Plus Real-Time PCR system (Applied Biosystems). Samples were normalised relative to the house-keeping gene, *Ywhaz* (Assay Mm.PT.39a.22214831, Integrated DNA Technologies).

### Protein extraction and western blot

Tissue sample were homogenised and quantified (Bradford assay; Sigma) as previously described^37^. Ten to 15 μg of untreated and treated *mdx* protein, and between 50% and 5% of C57BL/10 protein (see Fig. 2 for percentage loading for each tissue) were loaded onto 3-8% Tris-Acetate gels (Invitrogen). Protein gels were run for 110 minutes and blotted onto PVDF membrane. Membranes were probed for dystrophin using DYS1 (Novocastra) and loading control, vinculin (Sigma). Both primary antibodies were detected using IRDye 800CW goat-anti mouse IgG (Licor). Western blots were imaged (LiCOR Biosciences) and analysed using the Odyssey imaging system.

### Statistical Analysis

All values reported are mean ± standard error of the mean (SEM). Students T-test was used to compare dystrophin protein levels (western blot) in B-PMO unexercised and exercised cohorts. For MRI, Evans blue staining and RT-qPCR, all statistically significant values were determined using a one-way ANOVA followed by a Tukey post-hoc test. For MRI analysis, Games-Howell post-hoc test was also performed to correct for variance heterogeneity. Two-way ANOVAs were also performed on MRI data to determine interactions between exercise and mouse cohorts. The statistical significance for the diaphragm physiology was determined using Two-way repeated-measure ANOVA with Bonferroni’s post-hoc test.

## Additional Information

**How to cite this article**: Betts, C. A. *et al.* Implications for Cardiac Function Following Rescue of the Dystrophic Diaphragm in a Mouse Model of Duchenne Muscular Dystrophy. *Sci. Rep.* **5**, 11632; doi: 10.1038/srep11632 (2015).

## Supplementary Material

Supplementary Information

## Figures and Tables

**Table 1:** Cardiac function parameters measured by cine-MRI for *mdx,* C57BL/10 and B-PMO treated cohorts. All parameters are presented relative to body weight (BW).

	**1-way ANOVA**													**2-way ANOVA**
		**C57**^**unex**^	**SEM**	***mdx***^**unex**^	**SEM**	**B-PMO**^**unex**^	**SEM**	**C57**^**ex**^	**SEM**	***mdx***^**ex**^	**SEM**	**B-PMO**^**ex**^	**SEM**	**Interaction**
LV/BW	Average Mass	3.75	±0.20	3.43	±0.08	3.26	±0.09	4.04	±0.25	3.60	0.14	3.37	±0.09	N/S
	End Diastolic Volume	1.96	±0.14	1.77	±0.05	1.74	±0.06	2.03	±0.19	1.80	0.12	1.81	±0.08	N/S
	End Systolic Volume	0.74	±0.12	0.65	±0.08	0.72	±0.03	0.74	±0.18	0.87	0.15	0.72	±0.07	N/S
	Stroke Volume	1.22^ΔΔooo^	±0.04	1.12^o^	±0.04	1.02^**□□□^	±0.04	1.29^+ΔΔΔooox^	±0.04	0.93^***+□□□^	0.05	1.09^□^	±0.04	0.002
	Cardiac Output	0.52^+ΔΔoox^	±0.02	0.42^*□□^	±0.03	0.42^**□□^	±0.02	0.53^++ΔΔoox^	±0.02	0.41^□□**^	0.02	0.44^*□^	±0.01	N/S
LV%	Ejection Fraction	64	±2.7	64	±3.6	59	±0.9	66	±3.8	54	5.1	61	2.2	N/S
RV/BW	End Diastolic Volume	1.64	±0.04	1.53	±0.05	1.64	±0.08	1.66	±0.05	1.66	0.05	1.68	±0.09	N/S
	End Systolic Volume	0.45^ΔΔΔooo^	±0.02	0.55^oo^	±0.03	0.67^***□□^	±0.03	0.45^ΔΔooo^	±0.04	0.74^***□□□++^	0.04	0.67	±0.08	0.05
	Stroke Volume	1.18^+Δoo^	±0.04	0.98^*□^	±0.04	0.97^*□^	±0.06	1.21^+Δoox^	±0.06	0.92^**□□^	0.04	1.01^□^	±0.04	N/S
	Cardiac Output	0.50^+++ΔΔox^	±0.02	0.37^***□□^	±0.03	0.40^**□^	±0.02	0.50^++Δox^	±0.02	0.40^*□^	0.02	0.40^*□^	±0.01	N/S
RV%	Ejection Fraction	72^+ΔΔΔooo^	±1.01	64^*o^	±1.74	59^***□□^	±1.37	72^ΔΔoo^	±2.57	55^***+□□^	1.92	61.00	±3.35	0.02
BPM	Heart Rate	427	±15.4	373	±20.2	376	±13.2	410	±11.0	428	12.2	401	±8.6	0.04
Gram	Body Weight	32.4^Δo^	±0.78	32.4^Δo^	±1.20	35.9^*+□□□^	±0.66	29.6^ΔΔΔooo^	±0.72	35.9^*+□□□^	0.50	32.60	±0.79	<0.001

Note: The exceptions to this were the ejection fraction which is represented as a percentage and heart rate calculated as beats per minute (BPM). Weights are in grams. Statistical significance was determined using one- way ANOVA followed by Tukey post-hoc test (^TU^ANOVA). Games-Howell post-hoc test was also performed to correct for variance heterogeneity (^GH^ANOVA). Each cohort is compared to all other cohorts. ^*^significantly different to C57^unex^, ^+^significantly different to *mdx*^unex^, ^Δ^significantly different to B-PMO^unex^, ^□^significantly different to C57^ex^, ^o^significantly different to *mdx*^ex^ and ^x^significantly different to B-PMO^ex^. Number of symbols denotes significance i.e. ***p < 0.001, **p < 0.01, *p < 0.05. N = 10 for each cohort. Two-way ANOVA was also performed to show the interaction effect between exercise and the mouse groups (*mdx,* C57BL/10 and B-PMO treated mice).
