# Supplementary material for: Implications for Cardiac Function Following Rescue of the Dystrophic Diaphragm in a Mouse Model of Duchenne Muscular Dystrophy
Source: Sci Rep. 2015 Jun 26;5:11632. doi: 10.1038/srep11632 (PMC4586900; doi:10.1038/srep11632)
Supplement: Supplementary Information [file srep11632-s1.doc]

# Supplementary Information

**Title**

# Implications for Cardiac Function Following Rescue of the Dystrophic Diaphragm in a Mouse Model of Duchenne Muscular Dystrophy

# Authors

Corinne A. Betts^1^, Amer F. Saleh^2,3^, Carolyn A. Carr^1^, Sofia Muses^4^, Kim E Wells^4^, Suzan M. Hammond^1^, Caroline Godfrey^1^, Graham McClorey^1^, Caroline Woffindale^1^, Kieran Clarke^1^, Dominic J Wells^4^, Michael J. Gait^2^, Matthew J. A. Wood^1, *^

**Affiliations**

^1^ Department of Physiology, Anatomy and Genetics, University of Oxford, South Parks Road, Oxford, UK, OX1 3QX

^2^ Medical Research Council, Laboratory of Molecular Biology, Francis Crick Avenue, Cambridge, CB2 0QH, UK

^3^AstraZeneca R&D, Discovery Safety, Drug safety and Metabolism, Alderley Park, Macclesfield, SK10 4TG, UK

^4^Department of Comparative Biomedical Sciences, Royal Veterinary College, Royal College Street, London, NW1 0TU, UK


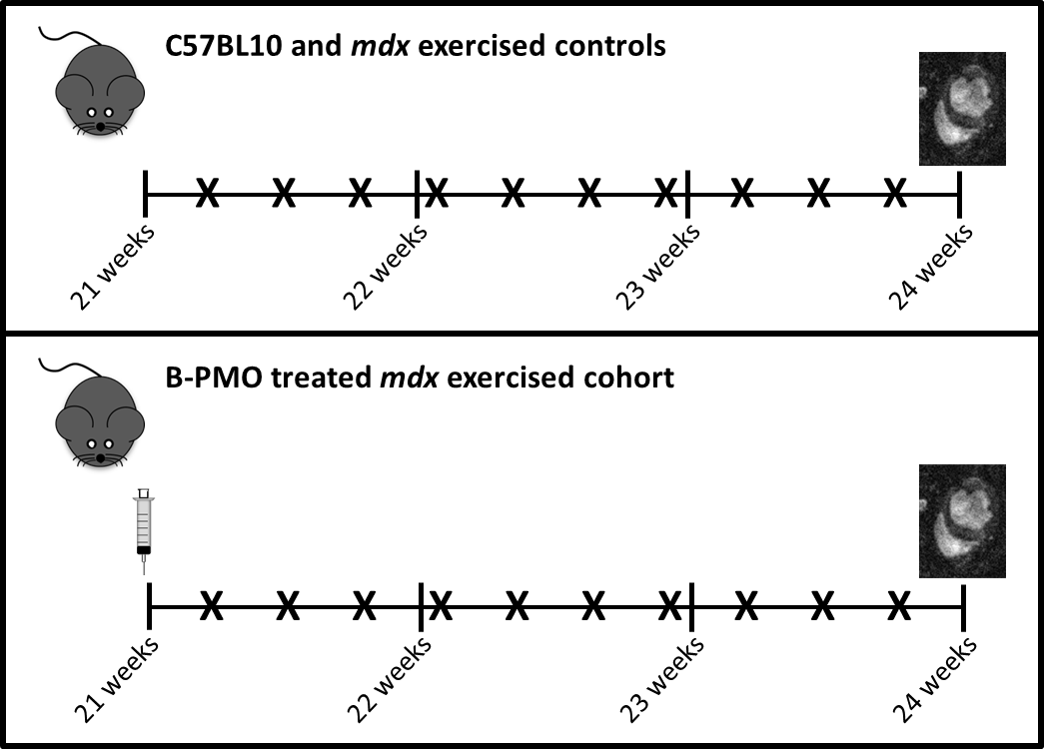


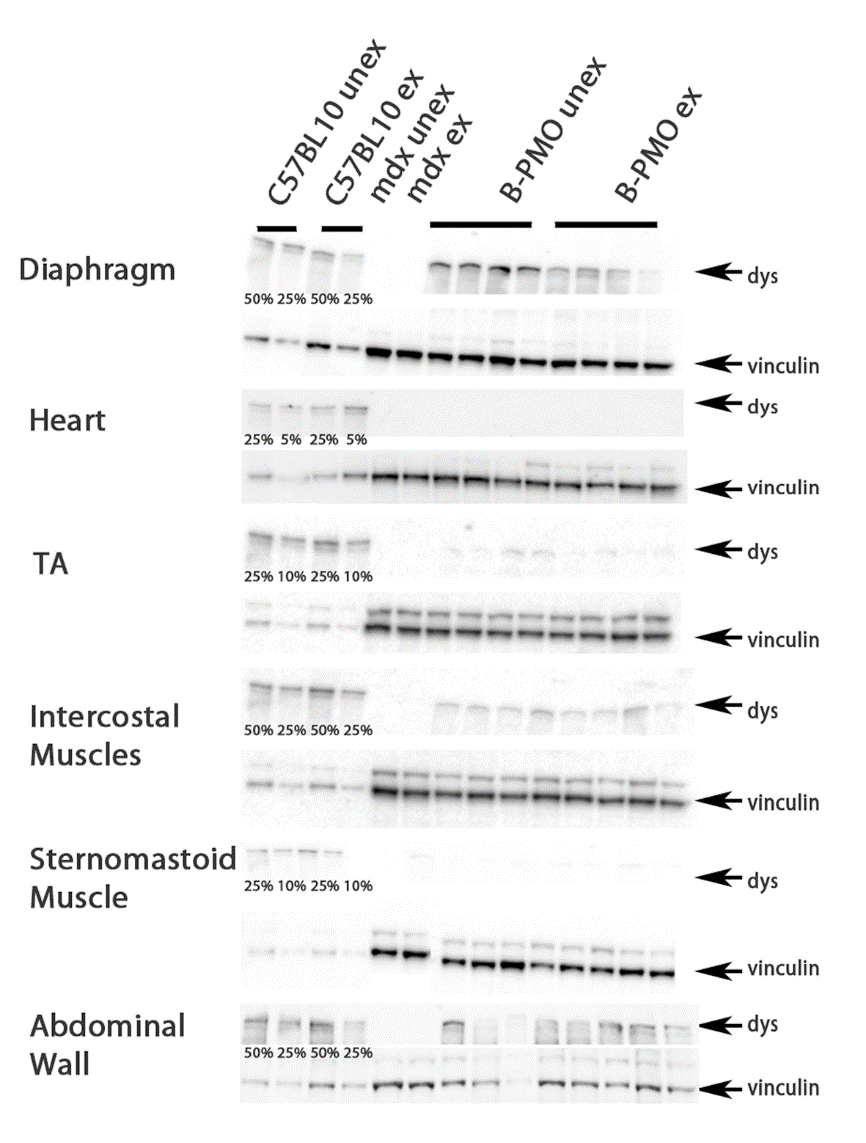


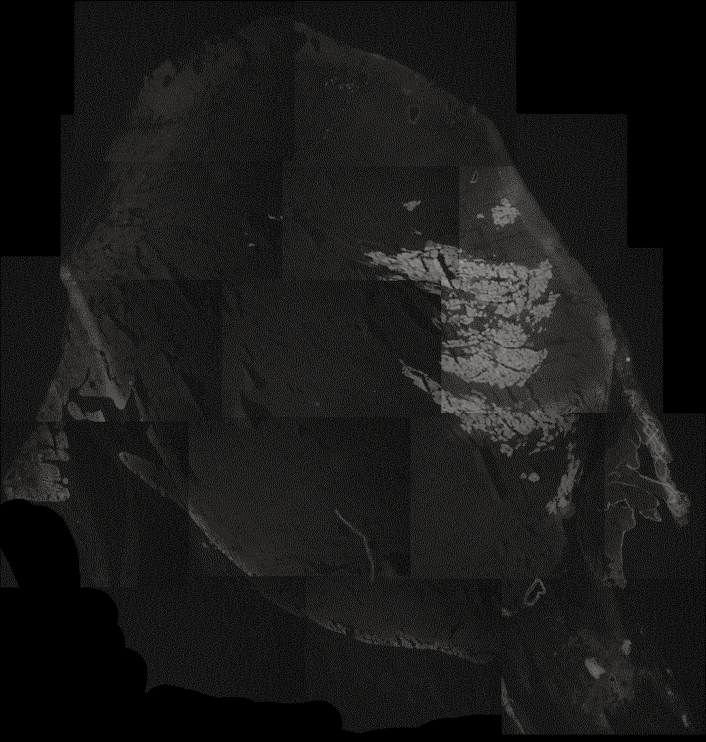


**Supplementary Methods**

The diaphragm physiology was conducted in the animal facility at the Royal Veterinary College under Home Office Licence and with the approval of the RVC Ethics committee. Seven 21-week old male *mdx* mice received a single dose of B-PMO (19mg/kg) via intraperitoneal administration, age and litter-match male *mdx* mice were used as untreated controls (n=8). Three weeks post injection, mice were killed by cervical dislocation and whole diaphragms including the attached ribs were placed in a wax based dissection dish containing cold Ringer’s buffer (137mM NaCl, 11mM Glucose, 5mM KCl, 2mM CaCl_2_, 1mM MgSO_4_, 1mM NaH_2_PO_4_, 24mM NaHCO_3_, pH7.4). Using entomology pins, the diaphragm was pinned and a 3-4mm strip was cut from the central tendon to the ribs along the orientation of the myofibres. A custom made 4mm wide coat hanger (0.40mm stainless steel, Scientific Wire Company) was tied to the ribs using 4-0 surgical braided silk thread and a custom made elongated steel s-hook (~4cm) was attached to the central tendon. The muscle was suspended between two platinum electrodes in a water jacketed organ bath containing Ringer’s solution, which was continuously perfused with 95%O_2_/5%CO_2_ and maintained at 28^o^C. The central tendon was anchored to the platform base (via the elongated S-hook), whilst the costal margin was attached to the lever arm of a 300C dual-mode servomoter transducer through the stainless steel coat hanger (Aurora Scientific, Aurora, Ontario, Canada). Electrical stimulation was generated through the two platinum electrodes, using square-wave pulses of 0.02 ms (701C stimulator; Aurora, Scientific). Data acquisition and control of the servomotor were conducted using a Lab-View-based DMC program (Dynamic muscle control and Data Acquisition; Aurora Scientific).

The diaphragm strip was left to equilibrate for 10 minutes prior to a warm-up protocol consisting of 3 submaximal contractions at 50Hz, 500 milliseconds duration; each contraction was delivered one minute apart. After defining the optimal voltage (16V), muscle length (Lo) was determined by increasing the muscle length until a maximal twitch force was achieved. The force-frequency relationship was evaluated by stimulating the muscle at different frequencies, delivered one minute apart (1, 10, 30, 40, 50, 80, 100, 120, 150 and 180 Hz). Maximal isometric force (Po) was determined from the plateau of the force-frequency curve. Resistance to eccentric contraction induced muscle damage was assessed by stimulating the diaphragm at 100 Hz for 500 milliseconds before lengthening the muscle by 10% of the Lo at a velocity of 0.5 Lo s-1 for a further 200 milliseconds, once the stimulation had ended the Lo returned at a rate of −0.5 Lo s-1. Between each contraction a 2-minute rest period was permitted to avoid muscle fatigue. A total of ten eccentric contractions were performed on each muscle. After each eccentric contraction the maximum isometric force was measured and expressed as a percentage of the initial maximum isometric force achieved at the start of the protocol, prior to the first eccentric contraction.

Diaphragms were removed from the water bath and the muscle carefully dissected away from the rib and central tendon. The muscle was blotted to remove excess liquid and immediately weighed prior to snap-freezing in isopentane pre-chilled in liquid nitrogen.

Muscle fibre cross-sectional area (CSA in cm^2^) was determined by using the following formula: muscle weight (g)/[dia fibre length (*L*f; cm) × 1.06 (g/cm^3^)]. Specific isometric force (N/cm^2^) was calculated by dividing the absolute force (N) at each stimulation frequency by diaphragm muscle fibre cross-sectional area. Statistical analysis for the force frequency and eccentric contraction studies was measured by a repeated measure two-way ANOVA followed by a Bonferroni post-hoc comparison. Statistical significance was defined as a value of p<0.05.

Supplementary Figure 1. Schematic illustrating the B-PMO administration and exercise regimen. At 21 weeks of age the treated cohorts received a single intraperitoneal administration of 19 mg/kg B-PMO. The exercise regime for the *mdx* and C57BL/10 cohorts (top) commenced the day after injection and continued for 3 weeks; 45 minutes exercise every 2 days. All mice underwent cine-MRI at 24 weeks of age. X symbolises exercise bouts, image of syringe symbolises administration of B-PMO.

Supplementary Figure 2. Western blots showing dystrophin protein in diaphragm, heart, *tibialis anterior* (TA), intercostal and sternomastoid muscles of B-PMO treated mice. 10-15 µg of protein was loaded and quantified relative to vinculin loading control. Percentage of C57BL/10 loading control indicated for each tissue. For quantification see Fig. 3b. N=4 for each cohort.

**Supplementary Figure 3.** Image of the exercised B-PMO treated mouse heart, which showed extensive Evans blue dye infiltration. 10X magnification images were taken and manually reconstructed to attain a consolidated image of the tissue section.

**Supplementary Methods.** Detailed description of diaphragm muscle physiology methods and materials.
